# Beneficial Effects of Tyrosol and Oleocanthal from Extra Virgin Olive Oil on Liver Health: Insights into Their Mechanisms of Action

**DOI:** 10.3390/biology13100760

**Published:** 2024-09-25

**Authors:** Daniela Gabbia

**Affiliations:** Department of Pharmaceutical and Pharmacological Sciences, University of Padova, 35131 Padova, Italy; daniela.gabbia@unipd.it; Tel.: +39-049-8275775

**Keywords:** tyrosol, oleocanthal, extra virgin olive oil, chronic liver disease, fibrosis, hepatocellular carcinoma, metabolic dysfunction-associated steatotic liver disease, metabolic dysfunction-associated steatohepatitis

## Abstract

**Simple Summary:**

Consumption of extra virgin olive oil (EVOO) is associated with many beneficial effects on human health as a result of its phenolic-derived compounds. This review explores the hepatoprotective properties and mechanisms of action of tyrosol and oleocanthal from extra virgin olive oil. Both compounds demonstrate antioxidant, anti-inflammatory, and modulatory effects on liver metabolism that improve chronic liver diseases, such as dysfunction-associated steatotic liver disease (MASLD) and liver fibrosis, and their progression to liver cancer. Mechanistic studies have suggested that these compounds exert their hepatoprotective effects through the regulation of multiple cellular pathways, including those involved in the antioxidant response and lipid metabolism. Understanding these mechanisms may provide valuable information for the development of therapeutic agents based on their chemical structures that are useful to improve liver health and prevent liver-related disorders.

**Abstract:**

The Mediterranean diet and consumption of EVOO are associated with multiple beneficial effects for human health, e.g. reduction in cardiovascular risk and mortality, improvement in the lipid profile, and the prevention of chronic diseases, such as cancers and neurodegenerative diseases. In EVOO, more than 30 different phenolic-derived compounds have been identified, representing one of the most promising bioactive classes in olive oil. This review explores the hepatoprotective properties of two of these compounds, tyrosol and oleocanthal, focusing on their mechanisms of action. Recent studies have shown that these compounds, which share a similar chemical structure with a hydroxyl group attached to an aromatic hydrocarbon ring, can potentially mitigate chronic liver diseases, such as MASLD and liver fibrosis, as well as their progression to liver cancer. Consequently, they deserve attention for future pharmacological drug development. In vitro and in vivo studies have suggested that these compounds exert these effects through the regulation of cellular pathways involved in antioxidant response, lipid metabolism, transcription factor activity, and NF-κB signaling. Understanding the mechanisms underlying the hepatoprotective properties of tyrosol and oleocanthal may provide valuable information for the development of therapeutic agents based on their chemical structures capable of targeting chronic liver diseases.

## 1. Introduction

The liver, the largest organ in the body, orchestrates many physiological processes, including nutrient metabolism, xenobiotic detoxification, bilirubin metabolism/excretion, energy, and lipid storage [1]. Many chronic diseases may affect the liver, causing a progressive deterioration of liver functions and leading to several symptoms, including jaundice, liver enlargement, and/or failure. The etiologies of chronic liver disease are highly variable, ranging from toxin exposure, prolonged alcohol abuse, and infection to autoimmune diseases and genetic and metabolic disorders, and differ in effect between women and men [1,2,3]. The final irreversible stages of all chronic liver diseases are advanced fibrosis and cirrhosis, which can evolve into hepatocellular carcinoma (HCC), and are the result of disruption of the liver architecture, formation of widespread nodules, vascular reorganization, neo-angiogenesis, and the deposition of extracellular matrix [2,4]. Although there has been little improvement from 2010 to 2019, cirrhosis and other chronic liver diseases continue to be one of the ten leading causes of death worldwide, according to the analysis of the Global Burden of Disease Study 2021 [5]. Furthermore, primary liver cancer, comprising HCC (75–85% of cases) and intrahepatic cholangiocarcinoma (10–15% of cases), represents the third leading cause of cancer-related deaths and the sixth most frequently diagnosed worldwide, with more than three-quarters of a million deaths and approximately 865,000 new diagnosed cases in 2022 [6].

Extra virgin olive oil (EVOO), one of the pillars of the Mediterranean diet, has been shown to exert several protective effects on the liver, including reducing liver steatosis, ballooning of hepatocytes, and fibrogenesis, as well as prevention of lipid peroxidation, among other benefits [7,8]. These effects have been attributed to its high levels of monounsaturated fatty acids, primarily oleic acid, as well as the content of many bioactive phenolic compounds, such as hydroxytyrosol and oleuropein. Recently, studies have been focused on the identification of the effects of other phenolic compounds, e.g. tyrosol and oleocanthal [9,10].

This review explores the hepatoprotective properties of tyrosol and oleocanthal from EVOO, focusing on their mechanisms of action. Both compounds demonstrated antioxidant, anti-inflammatory, and lipid metabolism-modulating effects, which could mitigate chronic liver diseases, such as metabolic-associated liver dysfunction, liver fibrosis, and hepatocellular carcinoma. This review focuses on the mechanisms of action through which these compounds may exert their hepatoprotective effects, exploring the cellular pathways they can regulate in hepatic cells.

To provide a state-of-the-art analysis of these studies, this review considered the literature published in PubMed, Scopus, and WOS up to April 2024, using the search terms “oleocanthal”, “tyrosol”, as well as “steatosis”, “metabolic dysfunction-associated steatotic liver disease”, “metabolic dysfunction-associated steatohepatitis”, “fibrosis”, or “liver cancer”.

## 2. The Phenolic Compounds of EVOO

EVOO phenolic compounds, with more than 30 different identified entities, are largely reported in EVOO composition and represent one of the most promising bioactive classes of compounds in olive oil [11,12,13]. They can be divided into different classes according to the presence in their chemical structure of specific functional groups: phenolic alcohols, characterized by a hydroxyl group attached to an aromatic hydrocarbon group; phenolic acids, subdivided into hydroxybenzoic acid derivatives and hydroxycinnamic acid derivatives; lignans, deriving from the condensation of aromatic aldehydes; secoiridoids, phenyl ethyl alcohol linked to elenolic acid or its derivatives; flavonoids, further divided into many subgroups all characterized by two benzene rings joined by a linear carbon chain [14]. The quantity and quality of these molecules in EVOO may vary depending on the cultivar, the harvesting time, the cultivation area, the extraction technology, and the subsequent production processes [15,16]. EVOO displays a greater content of phenolic compounds with respect to refined olive oils, mostly in the form of secoiridoids, e.g., oleuropein and oleocanthal, phenyl alcohols, e.g., tyrosol and hydroxytyrosol, as well as flavonoids, e.g., luteolin and apigenin [17,18].

**Figure 1:**
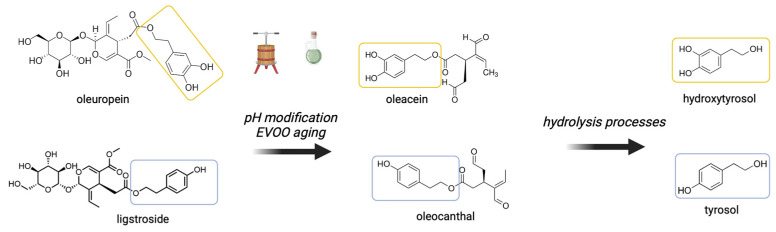
Secoiridoids and phenolic compounds formed during olive extraction and storage of EVOO. During aging and pH modification of EVOO, the secoiridoids oleuropein and ligstroside present in fresh-pressed olive oil are transformed into oleacein and oleocanthal. Following hydrolysis processes, they further break down into simpler molecules: hydroxytyrosol and tyrosol. Created with BioRender.com.

Secoiridoids, quite rare phenolic compounds among plant species, are abundant in Oleaceae species, particularly in the leaves and fruits of *Olea europaea* L., representing 70–90% of the total phenolic content [19]. Since they are quite insoluble in oil, only a small percentage of these compounds is present after the mechanical process of the extraction of EVOO with respect to other components, for example, fatty acids that account for almost 98% of its chemical composition [20]. Despite this low abundance, they confer the final sensory and organoleptic characteristics, such as bitterness, pungency, and stability [21,22,23,24,25]. The most common EVOO secoiridoids in olive drupes are oleuropein and ligstroside (Table 1) and their aglycones, accounting for approximately 90% of the phenolic compounds in EVOO [17]. During the extraction and storage of EVOO, these compounds undergo further processing due to the change in pH in the olive paste and the aging, generating other chemical species, for example, oleacein and oleocanthal (Figure 1) [19,26].

Among the phenyl alcohols, the two most represented species in EVOO are tyrosol and hydroxytyrosol (Table 1). During the storage of EVOO, these two phenols are released from complex phenolic compounds, such as secoiridoids, by hydrolysis (Figure 1) [27]. In addition, these two compounds have been shown to have many positive biological effects and have been associated with EVOO color and sensory qualities, even though their presence in oil is less than that of secoiridoids, as well as with the health-related and antioxidant properties of foods [28].

### 2.1. Oleocanthal

Oleocanthal, 2-(p-hydroxyphenyl)ethyl ester of (3S)-4-formyl-3-(2-oxoethyl)hex-4-enoic acid, was first identified in virgin olive oil by Montedoro in 1993 [29]. Chemically, it is the dialdehydic form of (−)deacetoxy-ligstroside aglycone, formed by tyrosol linked to elenolic acid, and its concentration in EVOO can vary greatly, ranging from 0.2 mg/kg to as high as 498 mg/kg of EVOO [9], depending on the cultivar, the maturity of the olive, the geographical area of cultivation, the growing conditions and agricultural techniques, as well as the EVOO methods of production, storage, and heating. Indeed, Italian EVOO has been reported as having one of the highest concentrations of oleocanthal, up to 191.8 ± 2.7 mg/kg of EVOO [30]. Although oleocanthal represents only 10% of the total polyphenol content of EVOO, it is responsible for the throat irritation and pungency characteristics of some EVOO [31]. This perception is a consequence of binding to a specific receptor, the transient receptor potential ankyrin 1 (TRPA1) [32].

Numerous studies reported in the literature suggest that oleocanthal may improve inflammation and oxidative stress, exerting beneficial effects on Alzheimer’s and neurodegenerative diseases [33,34], cancer, and rheumatic pathologies ([35] and refs. therein). Despite the evidence of multiple bioactive effects of oleocanthal, there is a lack of precise information on its intestinal permeability and in vivo bioavailability. Studies in animals demonstrated that secoiridoids and oleocanthal are poorly absorbed in the intestine with respect to other phenolic compounds of EVOO, e.g., hydroxytyrosol, and generally undergo phase I metabolism, generating mainly hydrated metabolites, but also hydrogenated and hydroxylated metabolites, then undergoing a glucuronidation reaction [36,37,38,39,40]. In rats, oleocanthal showed low absorption, with around 16% of the administered dose reaching the circulation, and high intestinal metabolism; in contrast, other evidence suggests higher absorption in humans [41]. In a recent study, no oleocanthal was detected in mouse plasma after oral administration, demonstrating its very short lifetime in vivo, whereas 13 metabolites were identified, specially oleocanthalic acid and tyrosol sulfate, that have been proposed as in vivo oleocanthal biomarkers and are likely to be responsible for biological effects attributed to oleocanthal administration [39].

### 2.2. Tyrosol and Other Phenolic Compounds

The main phenolic compounds of EVOO are tyrosol and hydroxytyrosol, both characterized by antioxidant activity. Tyrosol has been exploited as a therapeutic strategy for diabetes treatment due to its ability to prevent apoptosis of pancreatic β-cells through the modulation of JNK signaling and the improvement of endoplasmic reticulum (ER)-mediated stress, and for inflammatory lung diseases due to its ability to inhibit pro-inflammatory mediators by NF-κB-mediated inactivation [42,43]. Other studies reported a variety of other beneficial effects on various organs exerted by the phenolic compounds tyrosol and hydroxytyrosol, ranging from cardioprotective and neuroprotective to anticancer and endocrine effects [28]. In 2017, the European Food Safety Authority (EFSA) approved hydroxytyrosol as a novel food and recommended that 5 mg of hydroxytyrosol and its derivatives, for example, oleuropein and tyrosol, in olive oil should be consumed daily [44,45].

The biological effect of the phenolic compounds of EVOO is closely related to their pharmacokinetics and their metabolites. After oral administration, tyrosol is rapidly absorbed (in a percentage of 40–95% of the administered dose), metabolized in the liver into tyrosol-4-sulfate, and excreted renally within 8 h [46,47]. Generally, the conjugation of phenolic compounds, e.g., tyrosol, is low, although the administration of high doses has been shown to increase the conjugation rate as glucuronides [48]. Similar to tyrosol, even hydroxytyrosol was rapidly absorbed and distributed to organs, particularly the kidney, through which it is rapidly excreted as sulfoconjugates within 5 h after administration [49]. The urinary levels of tyrosol after a week of EVOO administration (25 mL/day) were lower than those obtained after a single administration of 50 mL, while urinary levels of hydroxytyrosol were similar, with an estimated recovery of hydroxytyrosol after short-term consumption greater than 100% [50]. Interestingly, in vitro studies observed that the reduction in the dopamine metabolite 3,4-dihidroxyphenyl-acetaldehyde (DOPAL), operated by the human hepatic alcohol dehydrogenase isoenzyme, leads to the formation of endogenous hydroxytyrosol and the metabolism of dietary tyrosine and other biogenic amines, such as tyramine, may represent an endogenous source of hydroxytyrosol [50,51]. A recent study by Alemán-Jiménez et al. demonstrated that the bioavailability of hydroxytyrosol is strongly affected by the food matrix in which it is incorporated [52]. Indeed, the highest plasma levels of hydroxytyrosol were obtained at 30 min after the oral administration of EVOO, whereas fortified flax oil, grapeseed oil, margarine, and pineapple juice showed no significant plasma levels of hydroxytyrosol, but a significant amount was excreted in the urine. This observation suggests that, although hydroxytyrosol was absorbed and followed a similar metabolism with different matrices, its pharmacokinetics and bioavailability are conditioned by the complex network of interactions occurring in the intestine, for example, the effects of microbiota, intestinal transporters, and in the first phase of the metabolism [52].

## 3. The Effects of Tyrosol and Oleocanthal on Steatotic Liver Disease

Steatotic liver disease (SLD), previously named fatty liver disease, encompasses a spectrum of liver diseases associated with other metabolic dysfunctions, e.g., diabetes, insulin resistance, and obesity [53,54,55]. Recently, the terms “non-alcoholic fatty liver disease” (NAFLD) and “non-alcoholic steatohepatitis” (NASH) were changed to “metabolic dysfunction-associated steatotic liver disease” (MASLD) and “metabolic dysfunction-associated steatohepatitis” (MASH) to avoid patients’ stigmatization, and the diagnostic criteria were also modified to better describe the complexity of the pathophysiological features [53,56]. MASLD development is characterized by the accumulation of fatty acids (FAs) into hepatocytes occurring through three main mechanisms: increased FA uptake, increased de novo lipogenesis and cholesterol accumulation in ER, and decreased FA oxidation [57,58]. In MASH, these pathological features were accompanied by increased inflammation and hepatocyte ballooning, which promote hepatic stellate cell (HSC) activation and prompt fibrogenesis, resulting in progression to irreversible stages of liver disease, like cirrhosis and HCC. The following sections report studies investigating the mechanisms of tyrosol and oleocanthal on lipid accumulation, oxidative stress, and inflammation, the main pathological features of MASLD/MASH.

### 3.1. The Effects of Tyrosol and Oleocanthal on Lipid Metabolism

**Figure 2:**
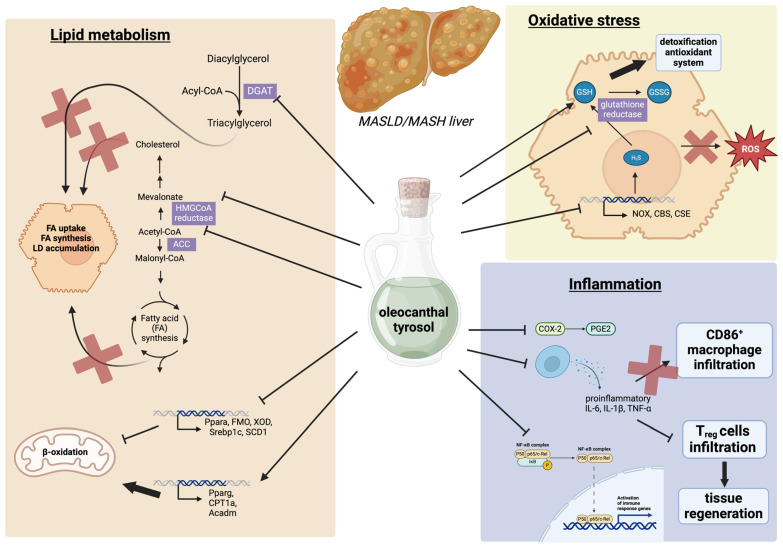
Main pathways regulated by tyrosol and oleocanthal involved in the hypothesized effects on MASLD/MASH improvement. Oleocanthal and tyrosol modulate a variety of pathological mechanisms, mainly related to three pathways: lipid metabolism, oxidative stress, and inflammation. In lipid metabolism, the two compounds appear to inhibit several processes, including fatty acid synthesis, uptake, and accumulation into hepatocytes. They are also able to activate antioxidant systems, leading to a decrease in intracellular ROS. Concurrently, they modulate hepatic inflammatory processes by reducing pro-inflammatory cytokines and macrophage infiltration and promoting T_reg_-mediated tissue regeneration. Abbreviations: FA—fatty acid, LD—lipid droplet, HMGCoA—3-hydroxy-3-methylglutaryl coenzyme A, ACC—acetyl-CoA carboxylase, DGAT—diacylglycerol acyltransferase, FMO—flavin monooxygenase, XOD—xanthine oxidase, Srebp1c—sterol regulatory element-binding protein 1c, CPT1—carnitine palmitoyltransferase 1, SCD—stearoyl-CoA Desaturase, IL—interleukin, TNF-α—tumor necrosis factor-α, NOX—NADPH oxidase, COX2—cycloxygenase, CBS—cystathionine β-synthase, CSE—cystathionine γ-lyase. Created with BioRender.com.

The effects of tyrosol and oleocanthal on steatotic hepatocytes have been investigated in many in vitro/in vivo studies that demonstrated their ability to improve the three main pathogenetic features of intracellular lipid accumulation: FA uptake, de novo lipogenesis, and decreased FA oxidation, as well as oxidative stress and inflammation through a variety of mechanisms (Figure 2). The phenolic compounds of EVOO, tyrosol and hydroxytyrosol, are capable of regulating lipid biosynthetic pathways. Indeed, in primary cultured rat hepatocytes, these two phenols showed reduced fatty acid synthesis and TG synthesis by inhibiting acetyl-CoA carboxylase (ACC), the enzyme catalyzing one step of de novo lipogenesis, and diacylglycerol acyltransferase (DGAT), respectively [59].

A study by Bur and colleagues suggested that tyrosol-containing olive leaf extracts may counteract or delay the complications of NAFLD by preventing the accumulation of lipids in hepatocytes, oxidative damage, and mitochondrial dysfunction and exerting a hypocholesterolemic effect due to the inhibition of 3-hydroxy 3-methylglutaryl coenzyme A reductase (HMG-CoA reductase) activity [60]. A similar antihyperlipidemic effect was previously observed in streptozotocin-induced diabetic rats treated with tyrosol for 45 days at a dose of 20 mg/kg body weight [61]. Indeed, in this animal model, tyrosol reduced hepatic HMG-CoA reductase and, in contrast, increased lipoprotein lipase and lecithin cholesterol acyltransferase in plasma.

Metabolomic analysis of mice with NAFLD induced by a high-fat diet and treated with tyrosol (supplementation of 0.025%, *w*/*w* of high-fat diet) for 16 weeks revealed that improved lipid homeostasis is related to hepatic increase in spermidine, taurine, linoleic acid, malic acid, and eicosapentaenoic acid (EPA) [10]. In tyrosol-treated NAFLD mice, *Pparα*, *Cpt1a*, and *Acadm* genes were upregulated, as well as PPARα and ACOX1 proteins, which were increased with respect to untreated mice, whereas gene expression of *Scd1* and *Srebp-1c* was reduced, confirming previous evidence demonstrating that tyrosol increases hepatic lipid oxidation and inhibits lipogenesis [10]. Another study observed a similar protective effect of 12-week tyrosol supplementation (23 mg/kg body weight) in female mice with metabolic syndrome induced by high-fructose diet administration [62]. Three phenylethanoid derivatives were studied, namely salidroside, tyrosol, and hydroxytyrosol. The study also analyzed their effect on intestinal microbiota modulation, but this effect was not observed for tyrosol, unlike the modulatory activity of salidroside and hydroxytyrosol. Furthermore, tyrosol shows a high-affinity binding for PPARα, similar to PPAR agonists, which may regulate hepatic fatty acid oxidation, insulin sensitivity, triglyceride metabolism, and adipogenesis, and there are ongoing clinical trials for MASH [63,64].

### 3.2. The Effect of Tyrosol and Oleocanthal on Oxidative Stress

The increase in hepatic oxidative stress is involved in MASLD progression to MASH and has been proposed as a target for preventing MASH development [65]. EVOO phenolic compounds are well known for their antioxidant activity. Supplementation with 5 mg/kg of body weight of tyrosol improves high-fat-diet-induced oxidative stress by modulating hepatic glutathione and lowering the GSH:GSSG ratio associated with liver injury by inactivating H_2_S biosynthesis [66]. This effect seems to be due to the upregulation of cystathionine β-synthase (CBS) and cystathionine γ-lyase (CSE) induced by tyrosol treatment. Another study demonstrated that phenols extracted from olive pomace (PEOP), including tyrosol, negatively regulate *Ppara* and positively regulate *Pparg* and *Cpt1*, thus stimulating mitochondrial β-oxidation and reducing FA uptake and lipid accumulation in steatotic hepatocytes. Furthermore, PEOP appears to improve endothelial and hepatic lipid-dependent oxidative imbalance by reducing reactive oxygen species (ROS) levels and NO release [67].

### 3.3. The Effects of Tyrosol and Oleocanthal on Hepatic Inflammation

Chronic low-grade inflammation is another key factor involved in MASLD/MASH progression, in which steatosis is accompanied by hepatocellular ballooning and lobular inflammation and is associated with an increased risk of fibrosis, which can evolve into cirrhosis and even HCC [65]. Tyrosol, in addition to improving steatosis, has been shown to ameliorate the inflammatory response triggered by a high-fat diet in mice by preventing up-regulation of JAK1 and STAT3 proteins and therefore of pro-inflammatory cytokines IL-6, tumor necrosis factor-α (TNF-α), and IL-10 [68]. Furthermore, tyrosol and hydroxytyrosol significantly decreased the increased hepatic expression of TNF-α, flavin monooxygenase 3 (FMO3), and xanthine oxidase (XOD) and the detrimental accumulation of hepatic trimethylamine N-oxide (TMAO) observed in high-fructose-fed mice [62].

Tyrosol has also been shown to affect AMPK phosphorylation, most probably by regulating the AMP/ATP cellular ratio or targeting SIRT1 deacetylase [59]. Olive leaf extract from a Sicilian cultivar, containing tyrosol and other phenolic compounds, has been demonstrated to improve lipid metabolism and decrease lipid droplets in hepatocytes, and concomitantly increase FABP-4, SIRT-1, and HO-1 expression and reduce the pro-inflammatory cytokines IL-1β and TNF-α [69]. Another study conducted on healthy subjects observed that the intake of polyphenol-enriched EVOO modulates different pathways, including activation of PPARs and AMPK/SIRT1/PGC1 cascade. Most of these changes are likely to be related to the anti-inflammatory, anticancer, and antioxidant properties of EVOO [70].

A recently published study demonstrated that tyrosol (10 mg/kg body weight) is capable of improving the three hallmarks of MASH—steatosis, inflammation, and fibrosis—exerting a modulatory activity in both innate and adaptive hepatic immune cell populations in MASH liver, by decreasing inflammatory foci and the accumulation of CD86^+^ macrophages, restoring the levels of CD4^+^ CD8^−^ T cells, and increasing those of CD4^+^ FoxP3^+^ Treg cells, which are involved in regenerative pathways [71]. This effect was accompanied by downregulation of the *NOX1*, *TGF-β1*, and *IL6* genes and positive effects on extrahepatic manifestations of MASH, e.g., fatigue and anxious behavior, which can affect the general quality of life of patients. Another study observed that the intake of virgin olive oil-based breakfast could switch the peripheral blood mononuclear cells toward a less inflammatory phenotype in patients with metabolic syndrome by transcriptionally modulating the expression of genes involved in inflammatory processes mediated by the nuclear factor kappa-light-chain-enhancer of activated B cells (NF-κB), activator protein-1 (AP-1), some cytokines, mitogen-activated protein kinases (MAPKs), or the arachidonic acid pathway [72]. In contrast, the administration of EVOO containing different amounts of phenolic compounds (2.92 mg/kg body weight) to female Ldlr−/−. Leiden mice with HFD-induced MASH failed to reduce liver inflammation and fibrosis and showed only improvement in weight gain and insulin sensitivity [73]. The authors of the study stated that these conflicting results may be due to the relatively high amounts of phenolic compounds administered to mice that resembled the highest chronic consumption at all feeding moments in humans. Therefore, these results warrant future long-term studies to examine the effects in humans of daily use of EVOO with a very high content of phenols.

### 3.4. Clinical Trials Assessing the Beneficial Effects of EVOO Consumption on MASLD

The beneficial effects exerted by healthy diets, like the Mediterranean one, in improving metabolic syndrome and reducing cardiovascular risks are well recognized and represent one of the most effective interventions in steatotic patients along with lifestyle changes [74,75,76,77]. Many studies investigated the effect of daily intake of EVOO on cardiovascular health and metabolic-associated diseases [78,79,80,81]. Table 2 summarizes the main clinical studies that evaluated the effects of EVOO consumption in patients with NAFLD. A prospective, open-label, non-randomized intervention study, including 44 untreated patients with NAFLD without fibrosis, evaluated the effect of consumption for 24 weeks of a traditional Mediterranean diet, which contains EVOO as the main fat in the diet. Adherence to the Mediterranean diet was estimated using MedDietScore, and improvement in liver imaging was evaluated by ultrasound elastography at the beginning and after 12 and 24 weeks. This study observed improvements in liver imaging, blood pressure, fasting glucose, glycated hemoglobin, C-reactive protein (CRP), and oxidized low-density lipoprotein levels after 24 weeks with respect to baseline values (NCT03203486, ClinicalTrials.gov accessed on 24 April 2024 and [82]). Similar results were obtained in the FliO study, another randomized clinical study evaluating the long-term effect of the Mediterranean diet on improving fatty liver in obese/overweight patients (NCT03183193, ClinicalTrials.gov accessed on 24 April 2024). The high antioxidant capacity of the Mediterranean diet supports its efficacy in the treatment of NAFLD in overweight or obese individuals, which induced a reduction in the fatty liver index from baseline after 6 months evaluated by ultrasound [83].

A single-blind randomized clinical trial comparing the effects of a Mediterranean diet with high EVOO content and of a low-fat diet on hepatic steatosis and insulin resistance in children with NAFLD observed that both induce an improvement, which is more prominent for the Mediterranean diet (NCT04415112, ClinicalTrials.gov accessed on 24 April 2024 and [84]). In these pediatric patients, adherence to the Mediterranean diet improved hepatic steatosis, insulin resistance, and levels of liver enzymes, particularly ALT, compared to the other group after 12 weeks of intervention.

In particular, a 2-month intervention study enrolling 23 subjects with steatosis daily supplemented with EVOO with high oleocanthal concentration observed a reduction in body weight, waist circumference, alanine transaminase, and hepatic steatosis associated with a decrease in some inflammatory cytokines, IL6, IL17, TNF-α, and IL1β [85]. In this study, NAFLD patients used four large spoons of oleocanthal-enriched EVOO (about 32 g of EVOO) during their main meals, e.g., at lunch and dinner, for a period of 60 days, without any other alteration in dietary habits or change in lifestyle. Thus, the improvement in liver index and liver steatosis was likely due to supplementation with EVOO in their diet.

In the APRIL study, 91 patients with obesity and prediabetes were advised to use EVOO enriched in oleocanthal and oleacein for 30 days. This study observed that supplementation with EVOO rich in oleocanthal and oleacein promoted body weight loss and improved fasting glucose, oxidative stress, and inflammatory status by modulating some inflammatory markers, e.g., CXCL1, IL-12p40, and IL-1RA. It also decreased IFN-γ, a cytokine whose increase is related to chronic inflammation, obesity-induced adipose tissue inflammation, and insulin resistance, and decreased glutathione reductase activity and total thiols [86]. Even though this study did not evaluate the effect on hepatic steatosis, the improvement in chronic inflammation and the observed weight loss may also be correlated with an improvement in hepatic steatosis. An ongoing clinical trial aims to assess the efficacy of polyphenol-enriched EVOO from different Sicilian olive cultivars in improving some parameters related to cardiovascular risk, metabolism, and inflammation in patients with metabolic syndrome, particularly lipidic and glycemic status, cytokine patterns, and endothelial dysfunction, and to establish the potential benefits on liver steatosis (NCT05282316, ClinicalTrials.gov accessed on 24 April 2024). The results of this study, expected at the end of 2024, will provide additional insights into the efficacy of EVOO compounds like oleocanthal and tyrosol on hepatic steatosis.

To date, no clinical trials have investigated the use of oleocanthal and tyrosol as single agents for the treatment of MASLD/MASH, although positive data on the efficacy of supplementation with EVOO enriched in phenolic compounds are likely to suggest the use of these compounds as single agents for the treatment of steatotic liver disease.

## 4. The Effects of Tyrosol and Oleocanthal on Hepatic Fibrosis

Liver fibrosis is the excessive accumulation of extracellular matrix proteins, including collagen, that occurs in most types of chronic liver disease, leading to liver scarring and impaired function [87]. Steatotic liver diseases, both MASLD and MetALD (in which MASLD is associated with alcohol consumption greater than 140 g/week and 210 g/week for females and males, respectively), alcohol abuse, chronic HBV and HCV viral hepatitis, and cholestatic and autoimmune liver disease are all potential causes of liver fibrosis that can develop into irreversible diseases, e.g., cirrhosis and liver cancer [88,89,90].

During fibrosis, injured hepatocytes release damage-associated patterns (DAMPs) and apoptotic bodies as a result of apoptosis, which activates quiescent hepatic stellate cells (HSCs) that release growth factors, e.g., transforming growth factor β (TGF-β), vascular endothelial growth factor (VEGF), and platelet-derived growth factor (PDGF), to promote the production of extracellular matrix and tissue regeneration [91].

### 4.1. Tyrosol and Oleocanthal Effects on Tissue Remodeling

Tissue remodeling in liver fibrosis involves significant changes in liver architecture due to excessive extracellular matrix (ECM) deposition, primarily collagen, driven by activated hepatic stellate cells. Dysfunctional ECM alters liver structure, disrupts hepatic lobules, and alters hepatocyte function. In addition, vascular changes increase vessel resistance and portal hypertension, leading to complications such as variceal bleeding and ascites. The remodeling process, accompanied by chronic inflammation, perpetuates ECM deposition and liver dysfunction, potentially progressing to cirrhosis, characterized by extensive scarring and regenerative nodules. This progression increases the risk of liver failure and HCC, promoting oncogenic changes. Consequently, tissue remodeling in hepatic fibrosis significantly impacts liver function and overall health, contributing to severe complications from liver disease. In TGF-β-activated LX2 cells, oleocanthal was able to reduce the expression of the two pro-fibrogenic markers α-SMA and collagen type I alpha 1 chain (COL1A1), as well as that of VEGFA and the metalloproteinases MMP2, MMP3, and MMP7 [92]. Oleocanthal (10 mg/kg body weight) was also shown to modulate the expression of microRNAs, miR-221-3p and miR-181-5p, involved in fibrogenesis suppression, in addition to upregulation of the two antifibrotic miRNAs, miR-29b-3p and miR-101b-3p, in mice with CCl_4_-induced liver fibrosis, suggesting multiple antifibrotic mechanisms [92].

### 4.2. Tyrosol and Oleocanthal Effects on Fibrosis-Induced Oxidative Stress

**Figure 3:**
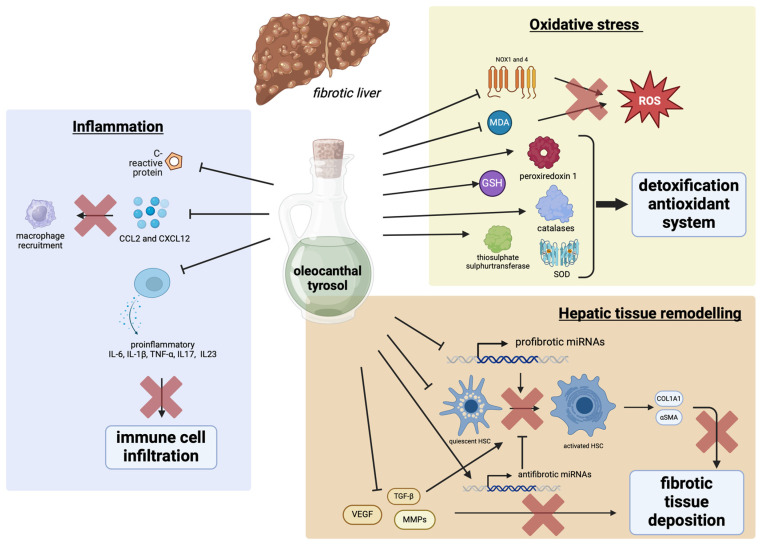
Main pathways regulated by tyrosol and oleocanthal involved in the hypothesized effects on liver fibrosis. This figure illustrates the multifaceted effects of oleocanthal and tyrosol on the fibrotic liver, focusing on the three pathogenetic pathways: inflammation, oxidative stress, and tissue remodeling. They are shown to inhibit macrophage recruitment and immune cell infiltration through the reduction in cytokine release and to activate antioxidant systems and detoxification processes by influencing factors like NQO1, MDA, and GSH. Meanwhile, oleocanthal and tyrosol can inhibit profibrotic miRNAs, activate HSC, and reduce fibrotic tissue deposition, affecting VEGF- and MMP-dependent pathways. Abbreviations: ROS—reactive oxygen species, IL—interleukin, TNF-α—tumor necrosis factor-α, NOX—NADPH oxidase, CCL2—C-C motif chemokine ligand, CXCL12—C-X-C motif chemokine 12, MDA—malonyl dialdehyde, TGF-β—transforming growth factor β, αSMA—α-smooth muscle actin, COL1A1—collagen type I alpha 1 chain, VEGF—vascular endothelial growth factor, MMPs—metalloproteinases. Created with BioRender.com.

As already mentioned, EVOO polyphenols may exert antioxidant and anti-inflammatory effects that are likely to counteract fibrosis progression (Figure 3). The administration of EVOO induced a lower level of malonyl dialdehyde (MDA) and of α-smooth muscle actin (α-SMA) and increased expression of peroxiredoxin-1 and thiosulphate sulphurtransferase, involved in the antioxidant and detoxification defense mechanisms of the liver [93]. In vivo studies have demonstrated the antifibrotic potential of tyrosol. A study conducted on a rat model of chronic liver damage induced with thioacetamide observed that tyrosol (20 mg/kg body weight) induced a histological improvement in inflammation, degeneration, and fibrosis due to the reduction in α-SMA and hepatocyte apoptosis, and the increase in glutathione (GSH) level, glutathione peroxidase (GSH.Px), and catalase (CAT) [94]. This study also confirmed the ability of tyrosol to reduce TNF-α, IL6, and TGF-β1. Another mechanism through which tyrosol can improve liver fibrosis is the modulation of two oxidative stress-related miRNAs, miR-181–5p and miR-29b-3p, which was reported in addition to the downregulation of the two NOX1 and NOX4 isoforms responsible for increased ROS production [95]. These two effects have been shown to act synergistically to improve liver fibrosis in a murine model obtained by CCL_4_ administration. In addition to the prompting effect on antioxidant cell defense, these studies suggest that both tyrosol and oleocanthal may improve fibrosis by acting as transcriptional regulators of miRNA expression. Oleocanthal was also effective in reducing NOX1 and NOX4, ROS-producing NADPH oxidases in hepatocytes [92]. Moreover, the administration of olive fruit pulp extract improved CCL_4_-induced hepatic fibrosis in mice by reducing AST, ALT, and ALP and restoring CAT, superoxide dismutase (SOD), and GPx normal levels, thus suggesting an effect on the enhancement of the ROS detoxification system and antioxidant enzyme activity [96].

### 4.3. Tyrosol and Oleocanthal Effects on Inflammation Induced by Fibrosis

Activated HSCs crosstalk with Kupffer cells that are stimulated to release proinflammatory cytokines and chemokines, e.g., TNF-α, IL1β, and chemokine (C-C motif) ligand 2 (CCL2), which sustain tissue inflammation recruiting monocytes and T cells from circulation, contributing to fibrosis progression. In this complex interplay between the different types of liver cells, increased reactive oxygen species (ROS) and oxidative stress play a central role in the progression of the disease [65]. An anti-inflammatory effect was observed due to the reduction in IL6, TNF-α, and C-reactive protein (CRP). A comparative study evaluating the effect of high intake of EVOO and corn oil (20% oil mixed in the standard rodent diet) in CCL_4_-induced fibrotic rats observed a less severe degree of liver damage after EVOO administration than corn oil, likely due to the presence of oleic acid and polyphenols in EVOO [93]. In addition to the reduction in oxidative stress due to transcriptional regulation of NOXs, oleocanthal also showed an inhibitory effect on the production of pro-inflammatory interleukins IL6, IL17, and IL23, and the two chemokines CCL2 and CXCL12 [92].

## 5. The Effects of Tyrosol and Oleocanthal on Liver Cancer

Primary liver cancer, mainly HCC and cholangiocarcinoma, has shown a steady worldwide increase in incidence and mortality over the years, and more than 1 million people will be expected to be affected annually by 2025, which represents a severe public health challenge [97,98,99]. Chronic liver diseases of different etiologies, e.g., viral infections, alcoholic hepatitis, and MASLD/MASH, can progress to liver cancer due to multiple altered pathogenetic mechanisms [99,100]. Recently, lipidomic studies observed dynamic changes in liver lipid composition, mainly triacylglycerols, phospholipids, sphingolipids, ceramides, FAs, and cholesterol, which are likely to be responsible for maintaining the development of primary liver cancer and could be exploited for future drug development [98].

**Figure 4:**
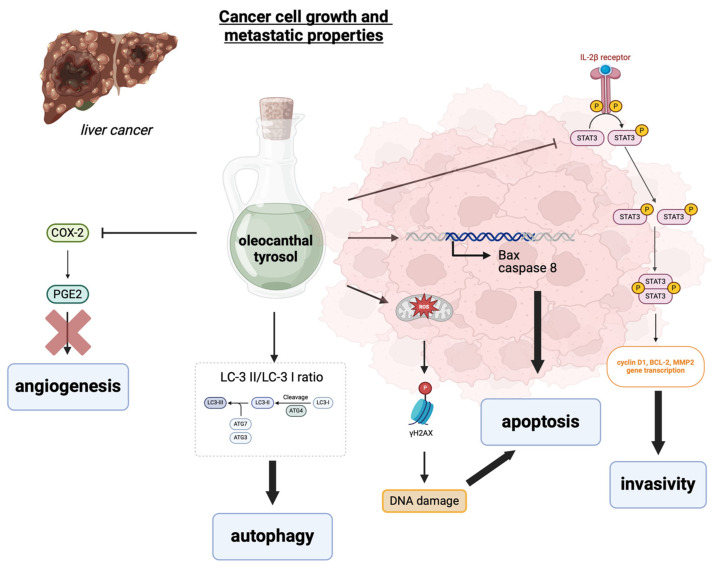
Main pathways regulated by tyrosol and oleocanthal involved in the hypothesized effects on HCC growth. Oleocanthal and tyrosol have multiple anticancer effects, including inhibiting angiogenesis through the inhibition of COX-2 and PGE2 activity, promoting apoptosis by affecting Bax and caspase 8 transcription, and autophagy, causing DNA damage, and reducing cancer cell invasiveness. Abbreviations: STAT3—signal transducer and activator of transcription 3, BCL-2—B-cell lymphoma 2, MMP2—metalloproteinase 2, LC-3—autophagy marker light chain 3, BAX—bcl-2-like protein 4, ROS—reactive oxygen species, PGE2—prostaglandin 2, γH2AX—γ phosphorylated form of the histone H2AX, COX2—cyclooxygenase 2. Created with BioRender.com.

As supported by numerous epidemiological studies, Mediterranean diet consumption is likely to protect against chronic diseases associated with inflammation, such as many different types of cancer [101]. The anticancer potential of EVOO phenolic compounds has also been exploited for liver cancer, particularly HCC (Figure 4).

Due to the anti-inflammatory activity exerted through the inhibition of COX2, oleocanthal counteracts the so-called “inflammogenesis of cancer” that has been demonstrated to sustain tumor growth, neo-angiogenesis, cancer invasion, and metastasis ([102] and refs. therein). Beauchamp et al. observed that an intake of up to 9 mg of oleocanthal per day derived from an EVOO intake of approximately 50 g corresponds to about 10% of the dose of ibuprofen used to relieve pain relief and may confer cardiovascular health benefits, acting similarly to aspirin inhibiting COX activity [103]. Recent studies have demonstrated that aspirin improves NAFLD and reduces the risk of HCC in NAFLD patients by inhibiting lipid biosynthesis and inflammation and increasing catabolism via activation of the PPARδ-AMPK-PGC-1α pathway [104,105]. Interestingly, oleocanthal has demonstrated chemoprevention for colon cancer by activating the AMPK pathway and inhibiting COX-2; therefore, it is likely to share the same effects with aspirin in the prevention of MASLD and HCC [106]. Oleocanthal has been shown to effectively inhibit HCC cell growth and metastatic capability by inhibiting STAT3 transcription factor activity. Thus, it downregulated STAT3 target genes known to promote HCC development and metastasis, e.g., the cell cycle protein Cyclin D1, the anti-apoptotic protein BCL-2, and the invasion-related metalloproteinase MMP2 [107]. In vitro, oleocanthal was able to effectively reduce cell growth and colony formation of a variety of HCC cell lines by inducing cell apoptosis, ROS production, and mitochondrial depolarization and increasing the expression of the γ phosphorylated form of the histone H2AX (γH2AX), a marker of DNA damage [108]. Another study observed that treatment with oleocanthal-enriched EVOO extracts induced the LC-3 II/LC-3 I ratio, an accepted marker of autophagic sequestration, a process acting as a “double-edged sword” on tumor progression, depending on tumor type and stage, and on HCC chemoresistance [109].

A recent study evaluated the antioxidant and anticancer activities of chitosan-lecithin-coated parthenolide/tyrosol nanoparticles (clPT-NP). This novel system decreased HepG2 cell survival, increasing cell apoptosis due to the upregulation of the two apoptotic genes, BAX and Cas-8, and the downregulation of the anti-apoptotic gene BCL-2 [110].

In addition to the interesting anticancer effects observed in in vitro studies on HCC, the translational value is partly limited by the fact that the concentrations used in in vitro studies (ranging from μg/mL to mg/mL) are difficult to achieve physiologically in humans, thus representing a challenge for the clinical evaluation of these compounds [111]. Furthermore, studies evaluating the anticancer mechanisms of tyrosol and olecanthal in liver cancer are few, and more research is needed to deepen our knowledge of the pharmacological effects of these two EVOO compounds in this type of cancer.

## 6. Conclusions

The Mediterranean diet and EVOO have been widely recognized for their beneficial effects on many chronic diseases and for reducing the risk of cardiovascular-related death. More recently, the EVOO phenolic components oleocanthal and tyrosol have been exploited both in vitro and in vivo on animals and humans to assess their ability to mitigate chronic liver diseases, such as MASLD/MASH, fibrosis, and HCC. Studies investigating their mechanism of action suggested that these compounds regulate multiple cellular pathways, including those involved in antioxidant response, lipid metabolism, and intracellular signaling (Table 3). The positive effects observed in metabolic-derived chronic liver diseases, such as MASLD and MASH, are of particular interest and deserve attention for the possible clinical application of these two compounds. Many recently published clinical studies demonstrated the efficacy of EVOO enriched with tyrosol and oleocanthal in patients with MASLD. This dietary approach could offer a safe and accessible way to promote liver health in conjunction with existing medical interventions and could be recommended to reduce the risk of developing chronic liver disease or slow its progression.

There are several limitations in current knowledge and important areas for future research regarding tyrosol and oleocanthal from extra virgin olive oil (EVOO) and their effects on liver health. To date, no clinical study has investigated the use of oleocanthal and tyrosol as single agents, although the preclinical evidence reported in this review encourages further clinical investigation to exploit the efficacy of a single agent in treating MASLD or MASH. The translational value of many in vitro studies is limited by the high concentrations of tyrosol and oleocanthal used, which are difficult to achieve physiologically in humans. This highlights the need for more pharmacokinetic studies to determine bioavailable doses and appropriate delivery methods. Future research should focus on controlled clinical trials that use these compounds individually to determine their efficacy and optimal dosing. Long-term studies are also needed to examine the effects of chronic consumption of high-phenol EVOO in humans, as some conflicting results have been observed in animal models.

Furthermore, EVOO supplementation may not be sufficient to treat fibrosis or HCC on its own. For this reason, studies exploring potential synergistic effects when combined with other treatments, as well as investigations into novel delivery systems to improve bioavailability, could provide valuable insights for developing these compounds as therapeutic agents for chronic liver diseases. Combination or supplementation therapy can be explored, as it has already been demonstrated with another compound of EVOO, hydroxytyrosol, which was successfully used to reduce systemic inflammation in pediatric patients with NASH in combination with vitamin E [112]. However, further research is crucial to fully elucidate the mechanisms of action of tyrosol and oleocanthal in the promotion of liver health and the prevention of liver-related disorders, particularly in the context of liver cancer. Current evidence from cell and animal models is promising, although human trials are necessary to support the future pharmacological development of these compounds as single agents to treat chronic liver diseases.

## Figures and Tables

**Table 1:** Chemical structures of some secoiridoids and phenyl alcohols of EVOO.

Compound Name	Chemical Structure
oleuropein	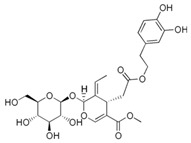
ligstroside	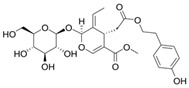
oleacein	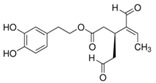
oleocanthal	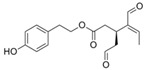
hydroxytyrosol	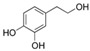
tyrosol	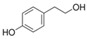

**Table 2:** Main clinical trials that evaluated the positive effects of EVOO and its phenolic compounds, including tyrosol and oleocanthal, on patients with NAFLD/MASLD.

Study Refs.	Study Type	Study Population	NAFLD Diagnosis	Treatment	Results
[82]	non-randomized, open-label, prospective intervention study	44 patients with untreated NAFLD without fibrosis (>18 years of age) and BMI > 25 kg/m^2^	abdominal ultrasonography and elastography stiffness (<75 kPa)	24 weeks of traditional Mediterranean diet, with increased EVOO, vegetables, whole grains, fruits, fish, and legumes	improved steatosis, decreased blood pressure, fasting glucose, and glycated hemoglobin, decreased C-reactive protein (CRP), and oxidized low-density lipoprotein levels
[83]	randomized study—FLiO: Fatty Liver in Obesity	98 overweight or obese patients with NAFLD (40–80 years of age)	abdominal ultrasonography	6 months of two energy-restricted diets (30% energy restriction): one standard restricted diet and MetDiet with increased natural antioxidant like EVOO	greater reduction in body weight, total fat mass, and liver fat compared to other type of diet after 6 months of follow-up in the Mediterranean diet group
[84]	single-blind randomized trial	45 pediatric patients with NAFLD (9–17 years of age)	abdominal ultrasonography	12 weeks of Mediterranean diet (high intake of EVOO, vegetables, fruits, cereals, nuts, and legumes) vs. low-fat diet	improved hepatic steatosis, insulin resistance, and levels of liver enzymes (ALT)
[85]	non-randomized, open-label, intervention study	23 subjects with metabolic syndrome and hepatic steatosis (18–70 years of age)	hepatic steatosis (by fatty liver index, FLI), abdominal fat distribution (by ultrasound)	4 large spoons daily of EVOO rich in oleocanthal (which corresponded to 32 g of EVOO) during their main meals, e.g., at lunch and dinner, for a period of 60 days.	reduction in body weight, waist circumference, body mass index, ALT and fatty liver index, IL6, IL17A, TNF-α, and IL-1β, and increasing IL10

**Table 3:** Selection of preclinical studies reporting the positive effect of oleocanthal and tyrosol as a single agent on liver health that reported their biological activities and mechanisms of action, specifically related to lipid metabolism, oxidative stress, inflammation, fibrosis, and liver cancer.

Compound	Biological Activity	Mechanism of Action	Study Type	Ref.
Tyrosol	Reduced fatty acid synthesis, de novo lipogenesis, and TG synthesis	Inhibition of acetyl-CoA carboxylase (ACC) and diacylglycerol acyltransferase (DGAT)	In vitro (primary cultured rat hepatocytes)	[59]
	Regulation of lipid metabolism	Increase in liver spermidine, taurine, linoleic acid, malic acid, and eicosapentaenoic acid Upregulation of *Pparα*, *Cpt1a*, *Acadm* Downregulation of *Scd1* and *Srebp-1c*	In vivo (high-fat-diet-fed mice)	[10]
	Increased lipid oxidation and inhibition of de novo lipogenesis	Reduction in total cholesterol insulin (INS), uric acid, low-density lipoprotein cholesterol (LDL-C), and aspartate aminotransferase (ALT), Reduction in TNF-α, flavin monooxygenase 3 (FMO3), and xanthine oxidase (XOD) Reduction in the detrimental accumulation of hepatic trimethylamine N-oxide (TMAO)	In vivo (high-fructose-fed mice)	[62]
	Antioxidant effect and inhibition of H_2_S biosynthesis	Modulation of hepatic glutathione, decrease in the GSH:GSSG ratio associated with liver injury, upregulation of cystathionine β-synthase (CBS) and cystathionine γ-lyase (CSE)	In vivo (high-fat-diet-fed mice)	[66]
	Reduced mitochondrial β-oxidation, FA uptake, and lipid accumulation	Downregulation of *Ppara* and upregulation of *Pparg* and *Cpt1*; decreased ROS production	In vitro (steatotic hepatocytes)	[67]
	Anti-inflammatory effect	Reduction in the up-regulation of JAK1 and STAT3 and decrease in IL-6, TNF-α, and IL-10	In vivo (high-fat-diet-fed mice)	[68]
	Improvement in inflammation, degeneration, and fibrosis	Reduction in α-SMA and hepatocyte apoptosis; increase in glutathione (GSH) level, glutathione peroxidase (GSH.Px), and catalase (CAT)	In vivo (thioacethamide-treated rats)	[94]
	Antifibrotic and antioxidant effect	Modulation of two oxidative-stress-related miR-181–5p and miR-29b-3p; downregulation of NOX1 and NOX4	In vivo (CCl_4_-treated fibrotic mice)	[95]
	Hypocholesterolemic effect	Inhibition of 3-hydroxy 3-methylglutaryl coenzyme A reductase (HMG-CoA reductase)	In vitro (steatotic HepG2 cells)	[60]
	Antihyperlipidemic effect	Inhibition of HMG-CoA reductase; increase in plasma lipoprotein lipase and lecithin cholesterol acyltransferase	In vivo (streptozotocin-induced diabetic rats)	[61]
	Improvement in the three hallmarks of MASH: steatosis, inflammation, and fibrosis	Decreased accumulation of CD86^+^ macrophages, restoration of levels of CD4^+^ CD8^−^ T cells, increase in CD4^+^ FoxP3^+^ Treg cells, involved in regenerative pathways downregulation of *NOX1*, *TGF-β1* and *IL6*	In vivo (high-fat-, high-fructose-diet-fed mice treated with CCL_4_)	[71]
Oleocanthal	Regulation of lipid metabolism Reduction of fatty acid synthesis and TG synthesis	Inhibition of acetyl-CoA carboxylase (ACC), enzyme that catalyzes one step of de novo lipogenesis, and diacylglycerol acyltransferase (DGAT)		
	Antifibrotic and antioxidant effect	Modulation of miR-221-3p and miR-181-5p, upregulation of antifibrotic miR-29b-3p and miR-101b-3p, downregulation of VEGFA, MMP2, MMP3, MMP7, NOX1, and NOX4	In vitro/in vivo (activated LX2 cells and CCl_4_-treated fibrotic mice)	[92]
	Anticancer effect	Activation of the AMPK pathway and inhibition of COX-2	In vitro (cancer cell model)	[106]
	Anticancer effect and inhibition of metastatic capacity	Inhibition of the STAT3 transcription pathway, downregulation of Cyclin D1, BCL-2, and MMP2	In vitro (HCC cell model)	[107]
	Anticancer effect	mitochondrial depolarization and increased expression of γ phosphorylated form of the histone H2AX (γH2AX),	In vitro (HCC cell model)	[108]
